# Antibodies and distortion of insulin and C-peptide results in patients with hypoglycaemia of unknown origin

**DOI:** 10.1016/j.clinme.2025.100542

**Published:** 2025-12-04

**Authors:** Adel A.A. Ismail

**Affiliations:** Consultant (RTD) in Clinical Biochemistry and Chemical Endocrinology, Mid-Yorkshire and Leeds teaching Hospitals, West Yorkshire, England

**Keywords:** Hypoglycaemia, Insulin autoimmune syndrome, Hirata disease, Interference in immunoassays, Factitious hypoglycaemia , Fictitious hypoglycaemia, Wrong isulin or C-peptide results

## Abstract

Two types of antibody could confuse the differential diagnosis of hypoglycaemia. One is insulin-binding autoantibodies (IAA), a double hit causing hypoglycaemia of insulin autoimmune syndrome (IAS, also known as Hirata’s disease) as well as distorting measurements of insulin and/or C-peptide. The clinical manifestations and initial endocrine results in patients with IAS would mimic and masquerade as other common pathologies, as well as factitious hypoglycaemia in adults, children and newborns. The second type is non-IAA autoantibodies which, if fortuitously/incidentally present in patients with hypoglycaemia, could interfere with insulin and/or C-peptide immunoassay measurements, also confusing differential diagnosis. Currently, testing for antibodies (all classes/subclasses) by simple method such as polyethylene glycol (PEG) is not usually included among first-line investigations of hypoglycaemia, causing delayed diagnosis or diagnostic misapplication. Detection of antibodies, irrespective of their nature, warrants cautious and careful differential diagnosis, averting hospitalisation and curtailing unnecessary and expensive investigations.

In adults, children and newborns, protracted and persistent hypoglycaemia is rare, but diagnostically a challenging condition. Common practice involves taking a blood sample during the hypoglycaemic phase for the measurement of insulin and C-peptide. The outcome of these results plays a major role in deciding ‘tentative diagnosis’, and further biochemical and radiological investigations follow to firmly establish diagnosis. Failure to test for antibodies could confuse such an approach because insulin-binding autoantibodies (IAA) of the insulin autoimmune syndrome (IAS), when present, could cause hypoglycaemia as well as distort insulin and/or C-peptide results, mimicking and masquerading as other pathologies and factitious hypoglycaemia.^1^, ^2^, ^3^, ^4^ Test for antibodies (all classes and subclasses) by polyethylene glycol (PEG) is simple, quick and informative, with trivial cost. Non-IAA autoantibodies (heterophile such as HAMA, anti-animal etc), if fortuitously present, would not cause hypoglycaemia *per se*, but may nevertheless interfere with insulin and/or C-peptide measurements, causing inaccurate and misleading results. Distinction between IAA and non-IAA autoantibodies could be made, if deemed necessary, by assessing insulin binding to endogenous antibodies *in vitro* (eg incubation with labelled insulin followed by electrophoresis / gel filtration chromatography to assess insulin bound to immunoglobulin binding). The purpose of this note is to emphasise the importance of including tests for antibodies, eg polyethylene glycol (PEG), in the early investigations of hypoglycaemia of unknown origin, in non-diabetic patients without previous exposure to insulin.

## The double hit effect of insulin-binding autoantibodies (IAA) on the clinical manifestations of hypoglycaemia and insulin measurements

Physiologically, signalling proteins on pancreatic ß-cells respond to a rise in blood sugar by the secretion of insulin (51 amino acids, molecular weight (MW) 5,808) and C-peptide (31 amino acids, MW 3,020) in equimolar amounts. Some 3% of intact proinsulin (86 amino acids, MW 9,390) and four partially processed split proinsulins are also produced (at 32–33 site and 65–66 site) and seep out into the blood prior to the final conversion of proinsulin to insulin and C-peptide by endopeptidases. Proinsulin has one-tenth of the insulin bioactivity and a much longer t_1/2_ than insulin. Proinsulin, as well as split proinsulins, structurally retain intact insulin and C-peptide.^5^

The presence of insulin autoantibodies (IAA) in the blood causes postprandial hyperglycaemia to persist for longer because insulin bound to IAA is physiologically inactive, incapable of lowering blood glucose. Persistent hyperglycaemia causes pancreatic secretion of insulin and C-peptide to continue until the IAA insulin-binding capacity is saturated, thus allowing the unbound/free active insulin concentration to rise and reduces blood glucose. At a concentration of ∼4.5–4.0 mmol/L (ie the glycaemic threshold), pancreatic secretion decreases/ceases.^2^^,^^3^

The IAA-binding characteristics and titre have crucial impact on the clinical manifestations of hypoglycaemia. Binding of insulin to IAA is reversible, influenced by two closely related but different and separate mechanisms, namely k**_on_** (mathematically calculable association rate), which determines the length of time insulin remains bound to IAA autoantibodies, and k**_off_** (mathematically calculable dissociation rate),^2^^,^^3^ which determines the speed of dissociation and release of insulin from IAA. The dynamics and rates are different; a significant k**_off_** would release free/unbound bioactive insulin in a concentration high enough to cause hypoglycaemia at different times, different durations and of varying severity manifested as spontaneous, postprandial, nocturnal or fasting hypoglycaemia (see ref.^2^^,^^3^ for detail). Hyperinsulinaemia also inhibits counter-regulatory mechanisms of glucose homeostasis (suppresses glycogenolysis, gluconeogenesis and ketogenesis), enhances C-peptide degradation and significantly increases in MCR causing a lower concentration in blood.^4^^,^^6^^,^^7^

Analytically, insulin bound to IAA is measured by immunoassay, producing high insulin results,^8^, ^9^, ^10^ which confuse interpretation because normally free unbound/bioactive insulin and C-peptide occur in blood. Furthermore, some immunoassays may additionally detect and quantitate insulin and/or C-peptide in proinsulin and split proinsulins. Different immunoassays are known to detect and quantitate all or only some of these entities,^8^, ^9^, ^10^, ^11^ causing considerable variabilities in both insulin and C-peptide results.

The double hit effect of IAA causing hypoglycaemia and erroneous insulin and C-peptide results would, with justification, confuse differential diagnosis, being clinically and biochemically capable of mimicking and masquerading as other pathologies and factitious hypoglycaemia.

## Insulin autoimmune syndrome (IAS) past and present

The first published case of IAS caused by IAA in adults was reported by Yukimasa Hirata and was written in Japanese^12^ in *J Jpn Diabetes Soc* in 1970 and subsequently in English in *Tohoku Journal of Experimental Medicine* in 1972.^13^ Transplacental transfer of IAA (IgG transfer is an active process involving numerous steps; affecting differently the four subclasses IgG1, IgG2, IgG3 and IgG4) during pregnancy from the mother to the fetus has occurred and been recently described, causing recurrent and protracted hypoglycaemia in newborns (ie secondary IAS).^14^^,^^15^ The IgG classes of IAA have a t_1/2_ of approximately 3–4 weeks. The first case of secondary IAS^16^ was reported and thoroughly investigated by Nakagawa in 1971 and published in 1973.^16^ A Japanese newborn (girl) who had protracted hypoglycaemia had secondary IAS with high IAA titre. Surprisingly, however, the baby’s 25-year-old mother had the same high titre of IAA as well as extremely high serum insulin levels, yet she was asymptomatic and did not suffer from any hypoglycaemia episodes. This case highlights (a) in the first few weeks of life, immunoglobulin in the neonate would be similar (but not identical) to the maternal profile, from whom different immunoglobulins subclasses have been transferred to the fetus. It would be therefore prudent or even necessary to investigate the mother of a newborn with refractory hypoglycaemia and (b) the lack of concordance between the kinetics and dynamics of k**_on_** and k**_off_** of IAA affecting maternal and newborn dysglycaemia *in vivo* and the measurement of insulin by immunoassays, which use high-affinity capture antibody quickly followed by quantitation with no/minimum dissociation from capture antibodies.

It is now recognised that IAS is common in individuals with human leukocyte antigen^2^^,^^3^ (HLA) types such as DR4, DRB1*0406, DRB1*0403, DQB1*0302, DQA1*0301, DRB1*0415, and DRB1*1301. The three major triggers of IAS in these predisposed individuals^2^^,^^3^ are viral infection/reinfection, eg mumps, rubella, Coxsackie B influenza, hepatitis C, chickenpox and measles. The innate immune response to infection/reinfection could increase the titre of autoantibodies exponentially (ie increase slowly at first followed by rapid rise, doubling every few hours). Drugs, specially those containing a sulphydryl (thiol) group or which produce this structure during metabolism, could also trigger IAS; examples are methimazole, pyritinol, glutathione methionine, 2-mercaptopropinoyl glycine, captopril, hydralazine, omeprazole, gliclazide and clopidogrel, and the OTC drugs α-lipoic acid (antioxidant). Biotin (vitamin B7 used to improve hair and nails) interferes with insulin and C-peptide immunoassays results. Patients with other autoimmune disorders are also known to be prone to developing IAS. Haematological disorders (monoclonal gammopathy of undetermined significance (MGUS)], or multiple myeloma) may produce immunoglobulins which could bind insulin, causing hypoglycaemia like IAS. However, IAS has occurred in some cases without any of the known risk factors.^17^

## Brief comments on interference in immunoassays by non-IAA

Terms such as ‘false positive’ indicate a disease presence when there is none, and ‘false negative’ the reverse. Clinicians are also generally aware that this may arise from the common/accepted statistical practice of establishing a ‘normal/reference’ range for individual parameters. The quoted reference range for each immunoassay test (as well as others) is obtained by using cut-off points (about 95%; +2 standard deviations) of a continuum obtained from ‘normal’ individuals. This statistical truncation delineates some false positive or false negative calculable data, expressed as predictive false positive and false negative rates using the well-established 2 × 2 contingency table.

Interference by autoantibodies in immunoassay is, however, different and should not be confused with results falling outside a normal established reference range produced by statistical truncation. The error rate in immunoassay tests is variable in magnitude, random in nature, insidious and unpredictable, and its impact on measurements differs from one immunoassay’s result to another. Inaccurate results caused by interference in immunoassay affects individuals who may fortuitously have endogenous immunoglobulin autoantibodies capable of interacting/interfering with reagents used in immunoassay measurements, namely two-monoclonal antibodies used as biological reagents and/or other components such as biotin, ruthenium etc.^18^, ^19^, ^20^, ^21^, ^22^, ^23^, ^24^ Each immunoassay ‘host’ presents his/her own endogenous autoantibody(s) as interacting ‘analytical reagents’. Because of the huge array and diversity of immunoglobulin autoantibodies that may be endogenously produced (around 10 billion), this form of potential interference and its magnitude is impossible to predict *a priori* and it would be time consuming, expensive and clinically unhelpful to subsequently establish its specific nature. The exact mechanism underpinning interference in immunoassay is described in detail elsewhere.^18^ Finally, it is important to emphasise that interpretation of immunoassay measurements must consider (a) the accuracy of the immunoassay test and (b) the incidence/prevalence of the disease for which the test is used. In patients in whom the incidence of the disease under consideration is high, the rate of false positive results is low. However, if the incidence of disease is low/unknown, the rate of false positive immunoassay results increases significantly, making follow-up confirmatory tests essential. For more details with examples, see ref.^25^^,^^26^

## Comments and conclusion

IAS is a complex and heterogeneous syndrome, caused by autoantibodies predominantly against insulin/proinsulin (common) or, less commonly, against the insulin receptors (this is a separate syndrome which may co-exist with other forms of IAS, for more detail, see ref).^2^^,^^3^ The incidence of IAS is undoubtedly much higher in East Asians than Caucasians, attributed to the prevalence of risk alleles in this population which is lower in Caucasians.^2^^,^^3^ It may be of interest, however, to point out that the first IAS case in a Caucasian^27^ (Norwegian) was published in the same year in which Hirata published the first Japanese case in English. Numerous IAS cases in Japanese were subsequently reported, with relatively few in Caucasians. For example, the first Dutch^28^ case (Caucasian) was described in 1996, while the first case in the UK was in a patient from East Asian background and appeared as an abstract in 2010; in a native Caucasian (Irish)^29^ in 2018 and in a Russian^30^ in 2020. The first case of IAS in an Italian (Caucasian) appeared in Sicily (population <5 million) in 2011, attributed to α-lipoic acid, a drug which was and still available over the counter worldwide. In less than 2 years, six more cases of IAS were identified by the same medical centre in Sicily.^31^ It may be therefore reasonable to assume that the exact incidence of IAS in Caucasians, though low, is not certain.

IAS in some patients was self-limiting after the resolution/discontinuation of the triggering factor and advice to eat small meals more frequently.^32^, ^33^ The spontaneous remission rate was reported to be very high in Japanese patients with IAS without any positive treatment and in whom the IAA is predominantly polyclonal. However, in Caucasians with monoclonal IAA, most patients respond to common therapies such as corticosteroid, acarbose and rituximab, however, in rare cases, hypoglycaemia persisted after all treatments including plasmapheresis.^34^

Diagnosis of IAS could only be established by testing for antibodies, irrespective of insulin and C-peptide results. In 2009, US endocrine society clinical practice guidelines added testing for autoantibodies among other first-line investigations; this was subsequently emphasised as mandatory^35^ in 2012. Tests such as polyethylene glycol (PEG 6000; 12.5% final concentration), which precipitate all classes and subclasses of antibodies, should be considered. It is therefore prudent or even mandatory to include/add a test such as PEG precipitation among first-line investigation of hypoglycaemia in all ages for establishing/excluding IAS diagnosis and other interfering antibodies. PEG precipitation is simple, inexpensive, informative and, in IAS patients with IAA, would made other investigations rather ancillary or unnecessary.

Finally, biochemical results received by clinicians are considered, with justification, to be accurate analytically, a reliable snapshot of information to support proper interpretation and clinical decision making.^4^ Ensuring accuracy and veracity of results may necessitate the addition of extra tests by the biochemistry laboratory such as PEG precipitation, serial doubling dilutions and repeat analysis using different analytical immunoassay platforms, even if not requested by clinicians.

## Highlights and take home message

(1) The presence of autoantibodies which may/may not bind insulin could distort initial immunoassay measurements of insulin and C-peptide; confuse interpretation and differential diagnosis of hypoglycaemia of unknown aetiology. Three follow-up affirmative tests are available in the routine biochemical lab.
(2) A PEG test is simple, inexpensive and informative, and should be included among first-line tests in patients with hypoglycaemia of unknown aetiology. The finding of a significant amount of autoantibodies (IAA or non-IAA) could make some investigations ancillary and enhance diagnosis, making long hospital admission unnecessary.

## Abbreviations

ISA: Insulin autoimmune syndrome, also known as Hirata disease

IAA: Insulin-binding autoantibodies

HAMA: Human anti-mouse antibody

## Funding

This research did not receive any specific grant from funding agencies in the public, commercial, or not-for-profit sectors.

## CRediT authorship contribution statement

**Adel A.A. Ismail:** Conceptualization, Writing – original draft, Writing – review & editing.

## Declaration of competing interest

The authors declare that they have no known competing financial interests or personal relationships that could have appeared to influence the work reported in this paper.
